# Novel Peptide Therapeutic Approaches for Cancer Treatment

**DOI:** 10.3390/cells10112908

**Published:** 2021-10-27

**Authors:** Caroline M. Li, Pouya Haratipour, Robert G. Lingeman, J. Jefferson P. Perry, Long Gu, Robert J. Hickey, Linda H. Malkas

**Affiliations:** 1 Department of Molecular & Cellular Biology, Beckman Research Institute of City of Hope, Duarte, CA 91010, USA; rlingeman@coh.org (R.G.L.); lgu@coh.org (L.G.); lmalkas@coh.org (L.H.M.); 2 Department of Molecular Medicine, Beckman Research Institute of City of Hope, Duarte, CA 91010, USA; pharatipour@coh.org (P.H.); rohickey@coh.org (R.J.H.); 3 Department of Molecular Diagnostics and Experimental Therapeutics, Beckman Research Institute of City of Hope, Duarte, CA 91010, USA; jperry@coh.org

**Keywords:** drug delivery, peptide therapeutic, covalent-based peptide inhibitors, PCNA

## Abstract

Peptides are increasingly being developed for use as therapeutics to treat many ailments, including cancer. Therapeutic peptides have the advantages of target specificity and low toxicity. The anticancer effects of a peptide can be the direct result of the peptide binding its intended target, or the peptide may be conjugated to a chemotherapy drug or radionuclide and used to target the agent to cancer cells. Peptides can be targeted to proteins on the cell surface, where the peptide–protein interaction can initiate internalization of the complex, or the peptide can be designed to directly cross the cell membrane. Peptides can induce cell death by numerous mechanisms including membrane disruption and subsequent necrosis, apoptosis, tumor angiogenesis inhibition, immune regulation, disruption of cell signaling pathways, cell cycle regulation, DNA repair pathways, or cell death pathways. Although using peptides as therapeutics has many advantages, peptides have the disadvantage of being easily degraded by proteases once administered and, depending on the mode of administration, often have difficulty being adsorbed into the blood stream. In this review, we discuss strategies recently developed to overcome these obstacles of peptide delivery and bioavailability. In addition, we present many examples of peptides developed to fight cancer.

## 1. Introduction

Rational drug design involves structural and functional studies to identify and disrupt targets important in cellular maintenance. Different approaches for drug development can include the use of small molecules, antibodies, short DNA aptamers, or peptides. Since the discovery of insulin in 1921, peptide drugs have been developed to treat a wide range of diseases that include cancer, immunological diseases, metabolic disorders, viral infections, cardiovascular diseases, and osteoporosis.

Modern biological research including large-scale genome sequencing and functional genomic studies greatly improved our understanding of malignancy. However, the advancement in our scientific knowledge has not yet been effectively translated into better cancer treatment. The majority of modern drug development efforts focus on a small group of 3000 druggable protein targets consisting of kinases and other enzymes, G protein-coupled receptors, ion channels, and nuclear hormone receptors [1]. This approach excludes more than 85% of the genome and is inadequate to the objective of finding a cure for all cancers. The advancement in peptide technology over the past decades is changing the drug discovery landscape. There are approximately 80 peptide drugs already in the market, more than 150 peptides in the clinical development stage, and another 400–600 peptides at the preclinical trial stage [2].

While the number of peptide drugs entering the market has increased significantly in the past decades, efficient delivery has limited their development. Pharmacologically active peptides are hard to formulate as drug products, as compared to small-molecules, due to the various challenges in administration and delivery of therapeutic peptides into cancer cells and tumor sites [2,3]. Typically, peptides drugs exhibit shorter circulation half-lives, lower cell permeability, and typically higher rates of enzymatic degradation. The oral delivery of peptides can have limitations, due to a number of factors that include enzymatic degradation and low absorption arising from metabolism by digestive enzymes or luminal microorganisms, the acidic environment of the gastrointestinal tract, the epithelial barrier of the small intestine, the unstirred water layer near the epithelial surface, and the various efflux systems [4,5,6]. Overcoming these difficulties would pave the way for developing more effective peptide therapeutics.

Here, we review recent strategies to improve peptide drug delivery issues, low bioavailability, and target selectivity through peptide modifications, with a particular focus on using peptide therapeutics for cancer treatment. We also describe how peptides are used to deliver drugs specifically to cancer cells and give examples of peptide targets that can lead to cancer cell death. Since therapeutic peptides have the advantage of high target specificity and low toxicity, overcoming its current limitations will lead to safer and more effective drugs.

## 2. Peptide Modifications for Enhanced Delivery and Stability

**Figure 1:**
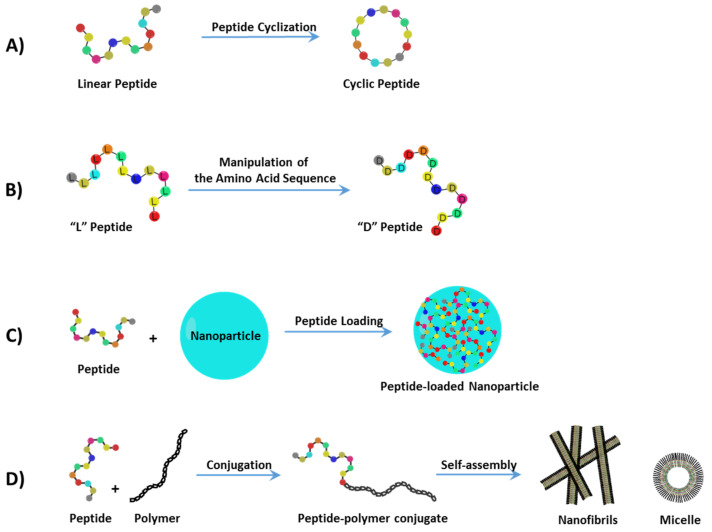
Current methods to enhance the half-life of peptide drugs and improve peptide drug delivery include (**A**) peptide cyclization, (**B**) manipulation of the amino acid sequence, (**C**) peptide-loaded nanoparticles, and (**D**) the conjugation of peptide drugs to natural or synthetic polymers.

In recent decades, great achievements have been made in the efficiency and selectivity of therapeutic peptide delivery [6,7,8,9]. The bioavailability and stability of therapeutic peptides have been increased due to the development of several formulation and delivery methods including prodrug approaches, direct chemical modifications, applying special drug delivery systems, co-administration of enzyme inhibitors, and absorption enhancers [10,11,12]. In this section, recent progress in the development of peptide delivery strategies (Figure 1) is discussed and their unique applications in cancer treatment are highlighted.

### 2.1. Peptide Cyclization

Free peptides are not systemically stable without modifications. Peptide cyclization (Figure 1A) is an example of a structural manipulation where the constrained geometries result in dramatically reduced proteolytic degradation by amino- and carboxypeptidases, due to the effects of masking both the *N*-terminal amino and C-terminal carboxyl groups [13,14,15]. An additional benefit of cyclization is that cyclic peptides adopt a limited number of conformations in solution, mainly β-turns, which can allow them to bind more efficiently to the active site of the desired target [13]. A successful example of peptide cyclization was reported by Hu et al.; it was observed that several cyclo-[Hcy^87^-Cys^96^] peptides of YAP (Yes-associated protein) were found to be drastically more potent than the linear YAP^84–100^ peptide in the disruption of the cancer-related TEAD–YAP protein complex [16]. Moreover, Duncan et al. identified a novel non-phosphorylated cyclic peptide inhibitor of Pin1, via screening of a phage display library of cyclic peptides, which binds and inhibits the peptidyl-proline isomerase activity of this enzyme [17]. Elevated levels of Pin1 have been observed in colon, breast, prostate oral squamous cell and lung cancers [18]. In another study, Lau et al. screened a library of random cyclic octapeptides using the ‘one-bead one-peptide’ technique and discovered that disulfide-cyclized cNGXGXXc peptide ligands (best hit: cNGQGEQc) promoted cell adhesion by targeting integrin α3β1 over-expressed in non-small lung cancer cells [15]. Cyclic peptides generally exhibit higher selectivity and stability compared to the corresponding linear precursors; yet, not all cyclization strategies will improve these properties.

### 2.2. Manipulation of the Amino Acid Sequence

The use of partially or fully substituted L-amino acids with D-amino acids (Figure 1B) provides another strategy to decrease proteolytic cleavage and lower immunogenicity. The replacement of L-amino acids with the corresponding D-amino acids at both termini or only of susceptible residues led to a stabilization of several peptides [12,19]. Octreotide, an FDA-approved octapeptide, is a well-known example of unnatural D-enantiomer modifications, which is used in the treatment of gastrointestinal tumors. Octreotide is the stable analogue of the parent peptide, somatostatin (which contains 14 natural amino acids). It has limited clinical application due to its very short plasma half-life (only a few minutes). However, octreotide was developed from somatostatin by replacement of all L-amino acids with D-amino acids and shortening the overall amino acid sequence to 8. This modification provided a significant increase in enzymatic stability, leading to higher plasma half-life of up to 1.5 h [20].

Recently, Zhao et al., has developed an antifouling peptide biosensor capable of detecting alpha-fetoprotein which is an important biomarker in many cancers, such as liver cancer. They have reported that although the CPPPPEKEKEKEK zwitterionic peptide (composed fully of natural L-amino acids) demonstrated excellent antifouling performances, enzymatic degradation limited its application in biological media. However, when the three unnatural D-amino acids were set at both terminals of the peptide (D-(cpp)PPEKEKE(kek)), its stability was enhanced dramatically which makes it reliable for long-term use in real biological samples [21]. The application of unnatural amino acids has been extended to other therapeutic peptides such as antimicrobial peptides (AMPs). In a comprehensive study by Lu et al., several cationic AMP Pep05 (KRLFKKLLKYLRKF) derivatives were synthesized by substituting L-amino acid residues with unnatural amino acids, such as L-homoarginine, 4-aminobutanoic acid (Aib), L-2,3-diaminopropionic acid (Dap), L-2,4-diaminobutanoic acid (Dab), D-lysine, D-arginine, and L-thienylalanine (Thi), and their antimicrobial properties were evaluated toward trypsin, plasma proteases, and secreted bacterial proteases. It was reported that the replacement of both L-lysine (K) in the *N*-terminus and L-phenylalanine (F) in the C-terminus of the parent Pep05 with unnatural Aib and Thi residues, respectively, afforded remarkably enhanced plasma stability and in vivo activity [22]. In addition, this study showed that although remarkable stability was achieved when all of the L-lysine and L-arginine residues were replaced by the corresponding D-amino acids (derivative DP06), the in vivo activity was minimal (probably due to the huge conformational changes compared to the parent peptide) and severe toxicity was obtained.

### 2.3. Peptide-Loaded Nanoparticles

Peptides can be formulated and delivered through nanostructured delivery systems (Figure 1C) as one of the strategies to improve the peptide absorption and circulation half-life. Polymeric nanoparticles (NPs) and silica NPs are the most common types of particles used for small molecule and peptide drug deliveries for cancer treatments [23,24]. In addition, the surface of NPs can be functionalized with distinct moieties, such as antibodies, proteins, peptides, vitamins, carbohydrates, and aptamers, all of which can be potentially used as targeted delivery carriers [25]. For example, Xie et al., developed the hollow mesoporous silica nanoparticles (HMSNs) for co-delivery of two melanoma-derived peptides with different hydrophobicity (HGP100_25–33_ and TRP2_180–188_). The HGP100 and TRP2 were loaded to HMSNs (named as HT@HMSNs) that were further enveloped with liposome, to form stable HTM@HMLBs. These HTM@HMLBs were then successfully applied to inhibit tumor growth and lung metastasis in murine melanoma models [26]. In another study, Qiao et al. developed pH-sensitive polymeric NPs for targeted delivery of RA-V and acid-triggered drug release. In this case, the natural plant cyclic hexapeptide RA-V, as a novel anticancer candidate, was loaded into hydrophobic cores of the poly(β-amino ester)s (PAE) NPs and efficiently delivered into tumor sites [27]. Natural polymers such as chitosan polysaccharides, composed of β-(1→4)-linked D-glucosamine and *N*-acetyl-D-glucosamine units, have been used extensively as a nanocarrier for oral peptide delivery [28]. Chitosan-based NPs exhibited excellent biocompatibility and gastrointestinal (GI) absorption and have been used to deliver anticancer agents [29,30]. With respect to therapeutic peptide delivery, Kanwar et al. demonstrated that lactoferrin polypeptide-loaded chitosan NPs can effectively activate apoptotic pathways in colon cancer and cancer stem cells [31]. Additionally, Gao et al. conjugated FQHPSF, a liver cancer-specific peptide, to chitosan NPs through a polyethylenimine (PEI) linker, which resulted in significant antitumor activity [32].

In addition, lipid nanoparticles including the class of Liquid Crystalline Lipid Na-noparticles (LCNP) provide protective reservoirs for encapsulated peptides. The properties of the LCNP can be described as an emergent type of drug delivery system suitable for peptide encapsulation and delivery. LCNP can be obtained by self-assembly of biocompatible lipids and co-lipids such as glycerol monooleate providing nanostructured lipid membrane medium for therapeutic peptide delivery with controlled release properties [33]. New possibilities for oral delivery are foreseen by the combination of LCNP and pH-sensitive biopolymers with mucoadhesive properties using positively charged *N*-arginine-modified chitosan and negatively charged alginate [34].

### 2.4. Conjugation of Peptide Drugs to Natural or Synthetic Polymers

Polymer/dendrimer–peptide conjugations (Figure 1D) have been used to improve bioavailability and stability by promoting nanoscale self-assemblies and reduced renal filtration by increasing the size of the peptide drug. Such conjugations include the covalent attachment of therapeutic peptides to: polyethylene glycol (PEG), poly(amidoamine) (PAMAM), poly(β-amino ester)s (PAE) and natural polysaccharides [35,36,37,38,39,40,41]. In addition to self-assemblies and targeted deliveries, peptide conjugates provide controlled drug release when using intelligent linkers under different intracellular stimuli. For example, esters and hydrazones are acid-labile groups that are hydrolyzed because of the low pH in endosomal compartments. The effects of different chemical linker properties on the toxicity of conjugated peptides were thoroughly reviewed by Bӧhme and Beck-Sickinger [42].

In one study, Kapoor et al., conjugated a 40 kDa PEG to the HVGGSSV peptide (discovered via phage-display technology from both Lewis lung carcinoma and GL261 tumors [43]) to effectively target Tax-Interacting Protein 1 (TIP1), a protein that was overexpressed in human-invasive breast cancer cells [44]. Liu et al., synthesized PKT-S-PEG, a peptide–dendrimer conjugate, by incorporating a cytotoxic peptide (KLAKLAK)_2_ (named KLAK), cell-penetrating peptide (TAT) and MMP2-sensitive peptide-PEG (S-PEG) onto a PAMAM dendrimer by one-pot-synthesis. PKT-S-PEG was found to penetrate deep into tumors resulting in efficient cell apoptosis. It was shown that the PEG chains were cleaved by MMP2 enzymes that are overexpressed in the microenvironment of glioblastoma tumors. Cleavage of the PEG chains reduced the size of dendrimers and exposed the cell-penetrating TAT peptide and KLAK ligand to tumor cells [45].

In a separate study by Qiao et al., KLAK was conjugated to poly(β-amino ester)s (PAE–KLAK) and it subsequently self-assembled to form micelle-like nanoparticles with pH-sensitive properties. These PAE-KLAK micelles displayed higher cytotoxicity against MCF-7 human breast cancer cells, as compared to free KLAK, due to their enhanced internalization by efficient cellular endocytosis. This resulted in increased mitochondrial membrane disruption that induced cellular apoptosis [40]. Furthermore, therapeutic peptides can be conjugated with peptide amphiphiles and consequently self-assembled into supramolecular nanostructures, such as micelles, twisted ribbons, and cylindrical nanofibers [46].

## 3. Treating Cancer with Cell-Targeting Peptide (CTP) and Cell-Permeable Peptide (CPP)

Peptides can be used to cause a therapeutic effect through direct binding with their target or through conjugation to therapeutics and use of the peptide for targeted delivery of the cargo [47,48]. Therapeutic peptides can be divided into two classifications, cell-targeting peptides (CTP) and cell-permeable peptides (CPP). CTPs bind to a molecular marker present on the targeted cell allowing delivery of conjugates to a particular cell type while sparing other cells from the often toxic effects of the therapeutic cargo. The CTP can exert its effect at the cell membrane, or from binding to its molecular target resulting in internalization of the peptide–therapeutic complex. Instead of binding to molecular markers on the cell surface, CPPs interact with charged components on the cell membrane, which then are internalized through various mechanisms. CPPs intended for anticancer therapy take advantage of the phenomenon that the outer cell membrane on cancer cells is negatively charged relative to normal cells thus enabling a positively charged peptide to preferentially target cancer cells [49,50,51].

### 3.1. Cell-Targeting Peptides

CTPs are designed to specifically bind to cell membrane proteins that are present in relative abundance on targeted cells compared to the rest of the cell population. The search for cell surface tumor markers has identified many proteins that are enriched in cancer cells versus normal cells, including integrins, epidermal growth factor receptor, G protein-coupled receptors, and other various receptors such as gonadotropin-releasing hormone receptor, vasoactive intestinal peptide receptors 1 and 2, neurotensin receptor 1, aminopeptidase N, and keratin 1, which are overexpressed in breast cancer [47,52,53,54,55,56,57]. Binding of CTPs to these receptors can result in activation or inhibition of signaling from the receptor and/or internalization of the peptide–receptor complex. To achieve a sufficient therapeutic window, a general rule is that at least a 3-fold increase in expression of the targeted protein should be present on cancer cells compared to normal cells. In addition, for peptides used to deliver anticancer agents, the expression level of the cell membrane target must be high enough to deliver a therapeutic dose [58,59].

One cell membrane protein target that has been identified is the epidermal growth factor receptor (EGFR), which is overexpressed in several tumors of epithelial origin including breast cancers of ductal or lobular origin. Antibody–drug conjugates (ADCs) have been effectively used to target EGFR in triple-negative breast cancer and have entered clinical trials [60]. Peptide–drug conjugates (PDCs) have many advantages over ADCs [47]. First, ADCs are large making it difficult to permeate far into tumors. Additionally, ADCs are often immunogenic despite efforts to humanize the antibody. Another problem is that ADCs often accumulate in excretory organs such as the kidneys and liver. In addition, antibody creation is expensive and time consuming. PDCs on the other hand are small and able to penetrate tumors with relative ease. They have low immunogenicity, are less likely to accumulate in excretory organs, are easy to synthesize, and are affordable to produce. 

To take advantage of the benefits that peptide therapeutics offer, CTPs are also being developed to target EGFR. GE11 (YHWYGYTPQNVI) is a peptide that was discovered by screening a phage display library for peptides with enriched binding to EGFR. GE11 binds to EGFR with a dissociation constant of 22 nM. This compares with a dissociation constant of 1–2 nM for the interaction of EGFR with EGF, one of the natural ligands for EGFR [61]. Importantly for a potential anticancer therapeutic, the mitogenic activation as a result of ligand binding to EGFR was much lower for GE11 compared to EGF. When proliferation of the human hepatoma cell line SMMC-7721 was measured in the presence of 1 µg/mL of GE11 or EGF over 48 h, treatment with GE11 resulted in an increase in proliferation of approximately 10% while treatment with EGF stimulated a 50% increase in growth. The EGFR/EGF complex is internalized upon receptor/ligand binding and likewise, GE11 results in internalization of the EGFR/GE11 complex. FITC-labeled GE11 was internalized into the high EGFR expressing SMMC-7721, but not internalized when the EGFR-negative cell line, K562, was treated with the labeled GE11. Furthermore, adding excess EGF or unlabeled GE11 to the cells resulted in decreased uptake of the FITC-labeled GE11. In vivo, intravenous delivery of I125-labeled GE11 resulted in accumulation of GE11 in the tumors of a mouse xenograft of the SMMC-7721 cell line. Four hours after tail vein injection of the radiolabeled GE11, the amount of radioactivity found in the tumor was greater than in any of the other tissues tested (blood, heart, liver, spleen, lung, kidney, and brain). These studies illustrate the ability of GE11 to effectively target EGFR expressing tumor cells. 

GE11 on its own does not cause appreciable cell death in the cells that it targets; however, many researchers have conjugated GE11 to polymers to form GE11-coated nanoparticles, micelles, and liposomes, which in turn are loaded with anticancer agents [62]. In this way, a cargo of therapeutic drugs can be delivered to EGFR expressing cells, avoiding much of the off-target toxicity associated with many drugs used to treat cancer. In addition, while uptake of nanoparticles is generally a result of passive diffusion, the presence of GE11 on the surface of nanoparticles results in increased active uptake of the coated nanoparticles via internalization of the EGFR–GE11–nanoparticle complex. GE11 has been used to target delivery of a wide range of agents including PC4, a photosensitive drug used in photodynamic therapy [63]; salinomycin, an inhibitor of breast cancer stem cells [64]; paclitaxel [65]; doxorubicin [66]; curcumin [67] and many other agents. One interesting approach sought to treat laryngeal cancer by conjugating GE11 to liposomes loaded with docetaxel. Treatment with docetaxel often results in upregulated expression of multidrug resistance genes, so the investigators also attached a small interfering RNA (siRNA) to the multidrug resistance gene, ABCG2, to the liposomes using electrostatic attraction. They found that by combining docetaxel with ABCG2-siRNA and using the GE11 to target the delivery, they were able to improve the antitumor efficacies and specificities in laryngeal cancer cells [68].

### 3.2. Cell-Penetrating Peptides

Unlike CTPs which target a specific molecular marker on the cell membrane, CPPs interact with the outer leaf of the cell membrane using primarily electrostatic forces. Once CPPs adhere to the membrane they translocate through the membrane to the interior of the cell by mechanisms that are not well understood. Preferential targeting of cancer cells with CPPs is achieved by taking advantage of the finding that the outer leaf of the cell membrane in cancer cells is more negatively charged than the membrane of healthy normal cells [49,50,51]. Consequentially, the composition of a CPP generally consists of a high percentage of basic amino acids (arginine, lysine, and histidine), which at physiological pH carry a positive charge on their side chain. The positively charged CPP is then able to electrostatically adhere to the negatively charged membrane. The source of negative charges on a cell membrane originate mainly from the phospholipid heads of the lipid bilayer but can also include other charged membrane components such as proteins. In addition to the charge of a CPP, the hydrophobicity of the peptide is also important. Many CPPs are amphipathic where the hydrophilic and hydrophobic residues influence the conformation of the peptide and cause the CPP to form α-helices or β-sheets upon binding to membrane phospholipids. CPPs forming α-helices have a very hydrophobic area on one face while the other face aggregates charged amino acid sidechains to foster electrostatic interactions. CPPs conformed to β-sheets have a stretch of hydrophilic amino acids and a stretch of hydrophobic amino acids, which facilitate adherence to the membrane and subsequent internalization. Like CTPs, CPPs can be used to deliver therapeutic cargo to target cells. CPPs have been used to deliver proteins, peptides, siRNAs, plasmid DNA and anticancer drugs. The cargo can be covalently conjugated to the CPP through chemical cross-linking or through cloning to create a CPP fusion product. Cargo with compatible charge to the CPP can also be non-covalently loaded onto the CPP using electrostatic forces. Small proteins, peptides and siRNA have been successfully delivered to targeted cells through non-covalent binding with CPPs [69,70]. The internalization mechanisms employed by CPPs are not well understood but broadly include endocytosis and direct translocation. However, predicting the mode of internalization of a CPP is difficult as it can change based on the concentration of the peptide, the cargo being carried, and the cells being targeted. In general, when CPPs are present on the membrane in high concentration they are internalized using energy-independent direct membrane translocation while at low concentration or when conjugated to cargo they use energy-dependent endocytosis to cross the membrane. There are, however, many examples in the literature where this general rule is not followed [71,72,73].

The promise of CPPs to deliver cargo to specific cell types while avoiding off-target effects has resulted in a skyrocketing of modified CPPs and the applications for their use. CPPsite 2.0 (https://webs.iiitd.edu.in/raghava/cppsite/index.html, accessed on 26 October 2021) is a database dedicated to compiling data on experimentally validated CPPs. At the time of writing there were 1855 entries in the CPPsite 2.0 database.

## 4. Possible Mechanisms of Therapeutic Peptides

The antitumor mechanism of therapeutic peptides is effectuated through many mechanisms including membrane disruption, apoptosis, tumor angiogenesis inhibition, immune regulation, or through inhibition of discrete internal targets [74,75]. The mechanism of action of many peptides involves the formation of pores or channels in the cell membrane. The pores can result in internalization of the peptide, but they can also be a means of cell death as a result of membrane disruption. The effect of a given peptide in this regard must be experimentally determined as the phenomenon is not well understood. However, several models have been proposed to explain the mechanics involved including the barrel-stave model, carpet model, and toroidal pore model which have been reviewed in several manuscripts [76,77]. In general, these models describe aggregation and arrangement of peptides to form channels into the cell membrane mediated by the amphipathic nature of the peptide and the phospholipid bilayer. The resulting conformational changes enable the peptide to enter the hydrophobic core of the membrane, where disruption of the membrane results in internalization of the peptide or cell breakage and necrosis as a result of dysregulated osmotic pressure. Significantly, cell death via membrane disruption can result regardless of growth rate or multidrug resistance mechanisms, conditions that often foil conventional chemotherapy approaches, while cationic residues in the peptide can enable preferential targeting of the peptide to the relatively anionic cell membrane of cancer cells. In addition to disrupting the cell membrane, peptides can also disrupt mitochondrial membrane potential resulting in the release of cytochrome c, activation of caspases, and induction of apoptosis [77,78,79].

Some peptides do not directly cause cancer cell death, but instead perturb the vascularization of the tumor to inhibit growth. These peptides inhibit vascular endothelial growth factor (VEGF) signaling, which normally signals for the neovascularization of tumors. By inhibiting VEGF signaling, the peptides prevent tumor growth and metastasis while having minimal effect on normal cells that have low requirements for neovascularization.

Another way that peptides can be used as an anticancer therapeutic is to elicit a tumor-specific immune response. In a recent example of this approach, a cell-penetrating peptide named cytosol localizing internalization peptide 6 (CLIP6) was conjugated to a model antigen, ovalbumin (OVA) [80]. CLIP6 is a CPP which passes through cell membranes exclusively by direct translocation and not by endocytosis, an important characteristic in that endocytosis often leads to endosome entrapment. The investigators found that the CLIP6–OVA complex entered cells effectively and resulted in an enhanced antigen uptake by antigen-presenting cells such as dendritic cells. In vivo, they found that the CLIP6–OVA complex when combined with CpG, an immune adjuvant, was able to trigger a strong antigenic-specific immune response in mice. Using the B16/OVA mouse model, which is a melanoma cancer model with cell surface expression of OVA, the researchers found that two out of six mice immunized with the CLIP6–OVA/CpG became tumor free while mice immunized with OVA or CLIP6–OVA all died within 31–39 days after inoculation with tumors. The results of this study illustrate a role for CPPs in the development of preventative or therapeutic cancer vaccines. 

Finally, therapeutic peptides can be used to target internal cell systems and structural proteins essential to signal transduction pathways, cell cycle regulation, DNA repair pathways or cell death pathways. A specific example of this is described below.

## 5. Rational of Targeting PCNA-Binding Proteins with a Peptide Derived from Cancer-Associated PCNA

This section describes in detail the rational towards designing a peptide drug with some considerations described in this review. The development of new peptide stability and delivery strategies is opening novel avenues for designing peptide therapeutics that can target key proteins in tumorigenesis. Proliferating cell nuclear antigen (PCNA) is one such target, and it is an essential protein involved in many processes including DNA replication, DNA repair, chromatin organization, transcription, sister chromatin cohesion and cell cycle control [81,82]. PCNA is highly expressed in cancer cells and is critical for cellular proliferation. Without PCNA, mice show embryonic lethality [83]. Patients with high levels of PCNA are linked with poor overall survival rates in breast [84,85], ampulla of Vater [86], non-small-cell lung [87], and pancreatic ductal adenocarcinoma [88] cancer.

A common strategy of designing peptide-based therapy is to disrupt protein–protein interactions (PPIs) with known protein binding sites. PCNA is a homotrimer that encircles DNA and acts as a platform that binds and coordinate proteins at the replication fork [89,90]. It is thought to interact and regulate over a hundred proteins [91] with various functions in the cell. Many, but not all, interacting proteins have conserved motifs that bind to PCNA, such as PIP-box (PCNA-interacting protein box), APIM (AlkB homologue 2 PCNA-interacting motif), and KA box (consisting of residues K-A-(A/L/I)-(A/L/Q)-x-x-(L/V)) [92,93]. The interdomain connector loop (IDCL) and a proximal hydrophobic patch on PCNA is one region where proteins have been indicated to interact [94,95], such as the PIP-box of p21 [96], peptide from the p66 subunit of DNA polymerase δ [94], and FEN1 [97].

To design a therapeutic peptide from PCNA that is specifically cytotoxic toward malignant cells, early studies focused on how PCNA in breast cancer cells was different. An isoform of PCNA was found in malignant breast epithelial cells and tissues but not non-malignant cells, using 2-dimensional PAGE experiments [98]. The cancer-associated isoform of PCNA (caPCNA) was likely from post-translational modifications [99] and not from genetic mutations or alternate splicing [100]. Cells from prostate cancer, hepatic carcinoma, high-grade prostatic intraepithelial neoplasia, and neuroblastoma [101,102,103] also had the unique PCNA isoform associated with cancer. Antibodies were developed using peptides derived from PCNA and the antibodies were screened for their ability to recognize the caPCNA isoform. Epitope screening studies led to the discovery of an 8-amino acid peptide, dubbed caPeptide, within the IDCL. Conjugating nine D-arginines linked by two cysteines to the *N*-terminus of the peptide led to the development of a cell-permeable peptide, R9-caPeptide, that was found to selectively inhibit malignant cancer growth instead of non-malignant and normal cells [104,105]. R9-caPeptide is an example of a cationic CPP for delivery to cancer cells.

**Figure 2:**
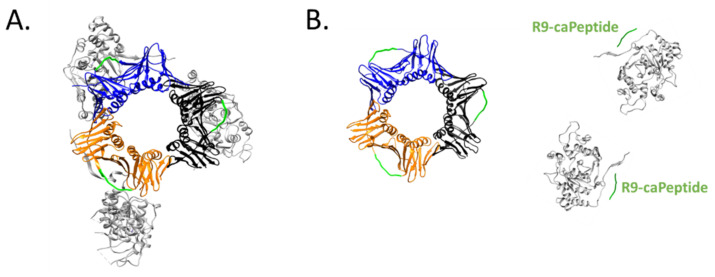
Schematic of targeting PCNA-binding proteins, such as FEN1. (**A**) Structure of the PCNA trimer (orange, blue, and black subunits) complexed to FEN1 (gray) [PDB ID: 1UL1 [97]]. The caPeptide region of PCNA (amino acids 126–133) is shown in green [PDB ID: 6FCM [108]]. (**B**) R9-caPeptide (labeled in dark green) binds to FEN1 to disrupt interactions with PCNA. The binding site of caPeptide-FEN1 shown in solution is theoretical. Images were made in the software Chimera [109].

R9-caPeptide was found to disrupt interaction of PCNA with its binding partners. Surface plasmon resonance studies showed disruption of PCNA with a peptide from FEN1 and the p66 subunit of DNA polymerase δ (POLD3) [105,106]. Additionally, caPeptide was found to interact with POLD3 [107]. Immunofluorescence microscopy studies showed that R9-caPeptide disrupted PCNA-LIG1 and PCNA-FEN1 interactions during DNA replication [105]. Figure 2 is a visualization of how R9-caPeptide prevents FEN1, an example of a PCNA-binding protein involved in DNA replication and repair, from binding to PCNA. 

Growth inhibition experiments showed that R9-caPeptide was cytotoxic in a dose-dependent manner to cancer cell lines derived from breast, lymphoma, neuroblastoma, and pancreas [88,104,105,107]. Treating malignant cancer cells with R9-caPeptide also caused stalled DNA replication forks, DNA damage, cell cycle arrest, and apoptosis. Validating the therapeutic potential, R9-caPeptide inhibited, in mice, xenograft tumor growth from triple negative breast cancer and from neuroblastoma cell lines [104,105]. Future directions may include peptide modifications described in Section 2.

## 6. Use of Covalent Warheads in Peptide-Based Therapeutics

Peptide-based therapeutics may provide a viable means to target PPIs, such as for PCNA, as perturbing interactions between partners that involve large surface areas can be a significant challenge for organic small molecule-based therapies. Peptides inhibitors to PPIs can be generated from peptide library screening strategies, or from structural studies that have characterized the PPI interface. Peptides identified by these approaches typically require further optimization of their drug-likeness through improving: affinity, selectivity, stability and cell permeability. An interesting direction to improve affinity and selectivity has been to add functional groups that can form covalent interactions with the protein target side chains in the binding site; such covalent coupling to the protein target provides a greatly enhanced potency of the inhibitor. Acrylamides and chloroacetamides have been extensively studied as moieties for targeting the cysteine side chain, and are being used in covalent targeting strategies for organic small molecules and peptide-based therapies. However, the ‘cysteinome’, proteins containing targetable cysteine residues, is somewhat limited due to the relatively rare occurrence of cysteine in a protein sequence. Thus, recent studies have been exploring potential chemistries to target other side chains and thereby extend the number of proteins that can be targeted through covalent-based approaches.

The discovery that aryl-sulfonyl fluorides and aryl-fluoro sulfates can act as covalent-warheads within peptide inhibitors notably expands the list of covalently targetable residues to now include lysine, tyrosine or histidine side chains. Aryl-fluoro sulfates may be of the strongest interest for therapeutic development, as they were confirmed to be cell permeable and stable in both aqueous buffer and plasma [110]. However, a concern from an initial characterization of this covalent warhead was an observed slow reaction rate, questioning the overall effectiveness of this warhead in forming covalent adducts within the cell. The initial study targeted a lysine residue that was relatively distant from the protein binding site, and encouragingly, a follow up study instead targeted a lysine within the peptide binding site, and rapid covalent adduct formation was readily observed [111]. Similar results were also observed in a separate study targeting human Mcl-1, using a BH3 substrate peptide for generating pro-apoptotic agent [112]. Thus, the rapid bond formation together with the cellular permeability, and stability being akin to that previously observed with acrylamides targeting cysteine, further suggests that aryl-fluoro sulfates may well expand the targetable residues beyond the cysteinome for novel therapeutic development. Other forms of potential covalent modifications strategies may be focused on the peptide substrate itself, adding stability and structure, to improve its binding characteristics. Cyclization of the peptide has been a common approach in this regard, and more recent studies have suggested other methods, including *N-locking*. A lactam bond is formed between the amino terminus and a glutamic residue at position 4, and this *N-lock* can nucleate helix formation within the peptide. *N-locking* can also be coupled with the covalent warhead strategy, as observed in BH3 peptides that were developed to target the antiapoptotic protein Bfl-1 to produce a peptide that was soluble in aqueous buffer and had low nanomolar affinity to its target [113]. Thus, these covalent-based approaches may provide novel avenues to develop peptide compounds with potentially more suitable ADME characteristics, and higher affinities and activities against their cellular targets.

## 7. Conclusions

The advancement of functional genomic studies is opening up many potential drug targets including transcription factors and structural proteins that are well validated but considered too difficult to manipulate by small molecules. Such potential targets include known oncogenes, such as the MYC family of proteins. It is well established that oncogenes are not the only specific changes in cancer cells. Cancer-specific changes can also occur in non-oncogenic structural proteins, such as PCNA, which acts as a “hub” in large cellular complexes which are essential for the growth and survival of all cancer cells. Peptides targeting such “hubs” are expected to be of a broader therapeutic spectrum than small molecules targeting specific signaling proteins. 

One major issue with the current target-based therapies is that drug resistance always arises in cancer cells through mutations of the target genes or activation of alternative signaling pathways. Unlike signaling components, many structural proteins have no alternative substitutes. Peptide-based agents often disrupt PPIs without binding to the target protein. Instead, they interact with potential binding partners of the target protein and bypass a major cause of drug resistance. Therefore, mimetic peptides acting as a decoy to a “hub” protein represents a therapeutic intervention less prone to development of resistance.

A major consideration in developing peptide therapeutics is addressing delivery problems that prevent adequate quantities of peptide to reach cancer cells. Several strategies have been described to increase the delivery and bioavailability of peptides, including peptide cyclization, employing natural or synthetic polymers, using D-amino acids, and using nanoparticles. To increase selectivity to malignant tissues and decrease toxicity, peptides can carry a load, such as a chemotherapy agent or another peptide that binds to proteins involved in tumorigenesis. Not only can peptide-based drugs disrupt essential PPIs utilized during the progression of cancer growth, but they can also disrupt membranes, affect the vascularization of the tumor, or induce an immune response that leads to cell death. The strategies and development of peptide therapeutics described here show promise in the laboratory with potential application to future cancer treatments.

## Data Availability

This review does not include analysis of new data.
